# Crystalline organic thin films for crystalline OLEDs (II): weak epitaxy growth of phenanthroimidazole derivatives†

**DOI:** 10.1039/d3ra03095d

**Published:** 2023-05-23

**Authors:** Dan Liu, Feng Zhu, Donghang Yan

**Affiliations:** a State Key Laboratory of Polymer Physics and Chemistry, Changchun Institute of Applied Chemistry, Chinese Academy of Sciences Changchun 130022 China zhufeng@ciac.ac.cn; b School of Applied Chemistry and Engineering, University of Science and Technology of China Hefei 230026 China

## Abstract

The ordered molecular arrangement of crystalline organic semiconductors facilitates high carrier mobility and light emission in organic light-emitting diode (OLED) devices. It has been demonstrated that the weak epitaxy growth (WEG) process is a valuable crystallization route for fabricating crystalline thin-film OLEDs (C-OLEDs). Recently, C-OLEDs based on crystalline thin films of phenanthroimidazole derivatives have exhibited excellent luminescent properties such as high photon output at low driving voltage and high power efficiency. Achieving effective control of organic crystalline thin film growth is crucial for the development of new C-OLEDs. Herein, we report the studies on morphology structure and growth behavior of the phenanthroimidazole derivative WEG thin films. The oriented growth of WEG crystalline thin films is determined by channeling and lattice matching between the inducing layer and active layer. Large-size and continuous WEG crystalline thin films can be obtained by controlling the growth conditions.

## Abstract 2

Study on morphology, structure and weak epitaxy growth behavior of 2FPPICz crystalline thin films provides an important basis for obtaining high-performance crystalline OLEDs.

## Graphical abstract

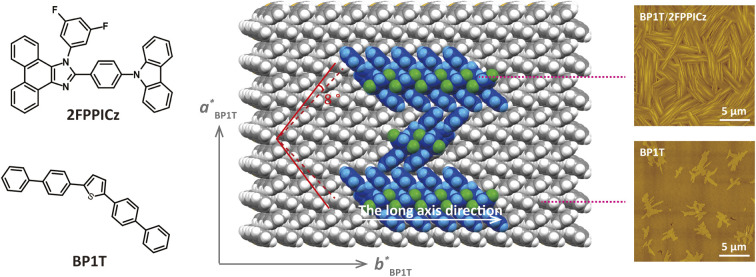

## Introduction

For organic optoelectronic devices, organic thin films are the core and have multiple functions such as carrier transport, light emission, and photoelectric conversion, which have a direct impact on device performance. Crystalline organic materials have drawn much interest in the field of optoelectronics due to their characteristics of low defects, highly ordered molecular arrangement, and thermal stability,^1–4^ which can help to enhance device performance, especially, the carrier mobility and light outcoupling efficiency in organic light-emitting diodes (OLEDs).^5–8^ However, the realization of highly-efficient crystalline luminescence has still faced many challenges. Recently crystalline thin-film OLEDs (C-OLEDs) fabricated by the weak epitaxy growth (WEG) method have realized efficient deep-blue luminescence adopting phenanthroimidazole derivative materials as the luminescence layers.^9–13^ Meanwhile, a kind of phenanthroimidazole derivative called 2-(4-(9*H*-carbazol-9-yl)phenyl)-1-(3,5-difluorophenyl)-1*H*-phenanthro[9,10-*d*]imidazole (2FPPICz) has been successfully applied as a crystalline thin film host material for high-performance C-OLEDs. Thanks to its high mobility and molecular orientation, in addition to the doping route,^13,14^ two kinds of high-performance blue C-OLEDs have been developed recently, namely the solid-solution C-OLED and “hot exciton” fluorescent nanoaggregate (HENA) sensitizing C-OLED. The HENA C-OLED has obtained a significantly improved performance with a maximum external quantum efficiency (EQE) of 9.14%.^15^ The solid-solution C-OLED in which the host matrix can induce the guest molecules orientation has achieved a maximum EQE of 6.5% beyond theoretical calculation value of 4.3%.^16^ The above-mentioned devices have achieved stronger blue emission and smaller driving voltage compared to all reported amorphous blue OLEDs with similar color coordinates. 2FPPICz crystalline thin films have become the basis for constructing high-performance C-OLEDs.

In our previous work, the crystal structure, lattice parameters and molecular orientation of phenanthroimidazole derivatives have been revealed in a model system of 2FPPICz thin film grown on a *p*-sexiphenyl (*p*-6P) double-layer film.^17^ It has been well proved that 2FPPICz molecules are arranged almost horizontally in the WEG crystalline films which is the favorable orientation for enhancing the light outcoupling efficiency. However, considering energy level matching with the 2FPPICz molecule, a material named 2,5-di([1,1′-biphenyl]-4-yl)thiophene (BP1T) was selected as the inducing and the hole transport layer and induced the epitaxy growth of 2FPPICz in the previously reported C-OLEDs.^12–16^ Therefore, the growth behavior, film structure, and morphology of 2FPPICz on BP1T are worthy of in-depth study, which will provide an important basis for obtaining high-quality large-size epitaxial films, optimizing the charge transport process, understanding the working principle of devices, and designing and developing new device structures.

In this work, the morphology and growth behavior of 2FPPICz crystalline thin films on BP1T layers has been investigated in detail by atomic force microscopy (AFM), X-ray diffraction (XRD), transmission electron microscopy (TEM) and selected area electron diffraction (SAED). The 2FPPICz molecules can perform weak epitaxy growth on BP1T monolayer and double-layer, but there is a smaller roughness and a larger width of 2FPPICz crystal strips on BP1T double-layer than that on BP1T monolayer, which is determined by the lattice mismatch between 2FPPICz and BP1T. Then, the 2FPPICz thin film growth process controlled by kinetics has been studied on the substrate of BP1T double-layer for the purpose of obtaining a deeper understanding of the WEG mechanism. These results are helpful for obtaining the knowledge on formation of low defects and large-size domain continuous crystalline thin films. These results will provide the key base for developing C-OLEDs in the future.

## Results and discussion

**Fig. 1:**
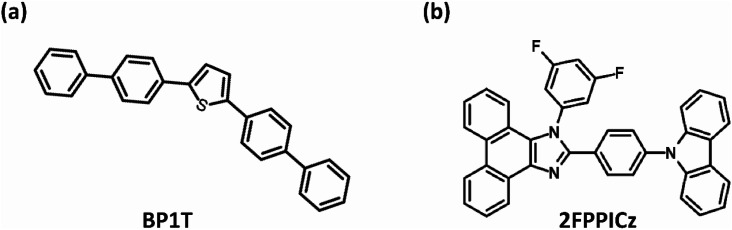
(a and b) Molecular structure of BP1T (a) and 2FPPICz (b).

**Fig. 2:**
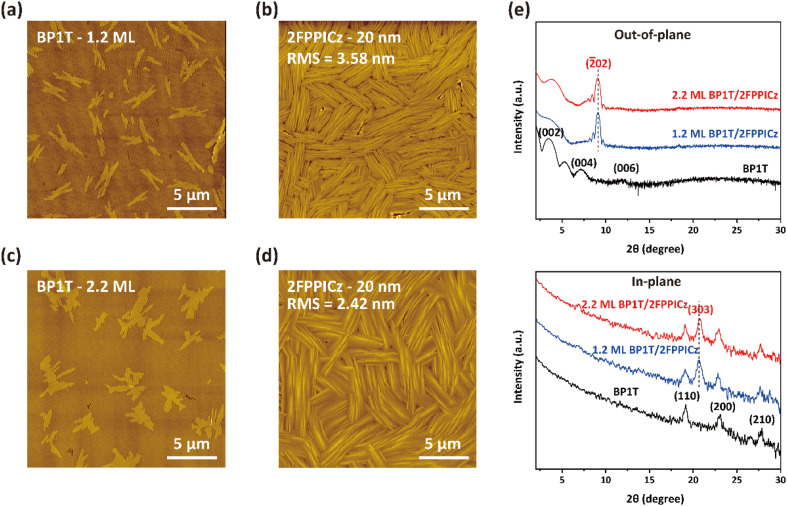
Morphologies and structures of BP1T/2FPPICz. (a) AFM image of 1.2-monolayer BP1T, (b) AFM image of 20 nm thick 2FPPICz thin film grown on 1.2-monolayer BP1T, (c) AFM image of 2.2-monolayer BP1T, and (d) AFM image of 20 nm thick 2FPPICz thin film deposited on 2.2-monolayer BP1T. (e) Out-of-plane and in-plane X-ray diffraction (XRD) patterns of BP1T double-layer thin film, 20 nm thick 2FPPICz thin film on BP1T monolayer and double-layer.

A suitable induction layer would be a large-size continuous thin film with a molecularly smooth surface.^10,18,19^ The molecular structures of BP1T and 2FPPICz are shown in Fig. 1. Fig. S1† shows the AFM morphology images of BP1T films grown at different substrate temperatures. As shown in Fig. S1a–d,† it can be seen that the size of the BP1T crystalline domain gradually increases as the temperature increases, and the size of the crystalline domain is larger when the substrate temperature is 112 °C. While the crystalline domain of the film is larger, the grain boundaries and defects will be fewer. Thus, we hope to obtain a film with a larger crystalline domain size. However, when thicker BP1T thin films are grown at a substrate temperature of 112 °C, we observed annealing of BP1T films as shown in Fig. S1e and f.† It can be seen that 112 °C is not conducive to the growth of continuous BP1T films. Therefore, we chose a substrate temperature of 102 °C to grow the BP1T film. Fig. S2† shows the AFM images of BP1T thin film with different thicknesses deposited on Si/SiO_2_ substrate at the substrate temperature of 102 °C. It can be seen that the BP1T molecules grow layer by layer to form a continuous thin film, which can provide a flat surface for the subsequent growth of organic semiconductors. Therefore, it can be used as the induction layer for the WEG process. Then we respectively prepared 20 nm thick 2FPPICz thin films on BP1T monolayer (Fig. 2a) and double-layer (Fig. 2c) substrate. The 2FPPICz molecules can perform an epitaxy growth to form strip-like crystals on both BP1T monolayer and double-layer thin film as described in Fig. 2b and d. However, the root-mean square (RMS) roughness of the 2FPPICz thin film on BP1T monolayer is evidently larger than that on double-layer. In addition, by measuring the width of the strip-like 2FPPICz crystalline domains, it is revealed that the average width of the 2FPPICz crystal on the BP1T monolayer was 200–300 nm (Fig. S3a†), which was smaller than that of 500–600 nm on the double-layer (Fig. S3b†). The length of the 2FPPICz crystals on both BP1T monolayer and double-layer was consistent with the single domain size of the inducing layer. In order to get more structural information, we carried out X-ray diffraction (XRD) measurements on the thin film as displayed in Fig. 2e. The out-of-plane diffraction peak associated with the BP1T layer corresponds to the (00*l*) reflection (CCDC number of BP1T is 1105416).^20^ Thus, the BP1T molecules are standing upright on the substrate. The out-of-plane diffraction peak (2*θ* = 9.14°) of 20 nm thick 2FPPICz thin film on BP1T monolayer (blue solid line) and double-layer (red solid line) was the same, which can be assigned to 2FPPICz (2̄02) reflection. But in the in-plane XRD measurement, the 2FPPICz (303) diffraction peak on the BP1T double-layer has a slight shift compared to that on the BP1T monolayer. According to the Bragg equation, the interplanar spacing of 2FPPICz (303) diffraction on the BP1T monolayer and double-layer can be calculated as 4.30 Å and 4.27 Å, respectively. Therefore, on the BP1T double-layer, the value is closer to 4.26 Å which is the interplanar spacing of (303) diffraction in the single crystal data (CCDC number is 2106623).^17^

**Fig. 3:**
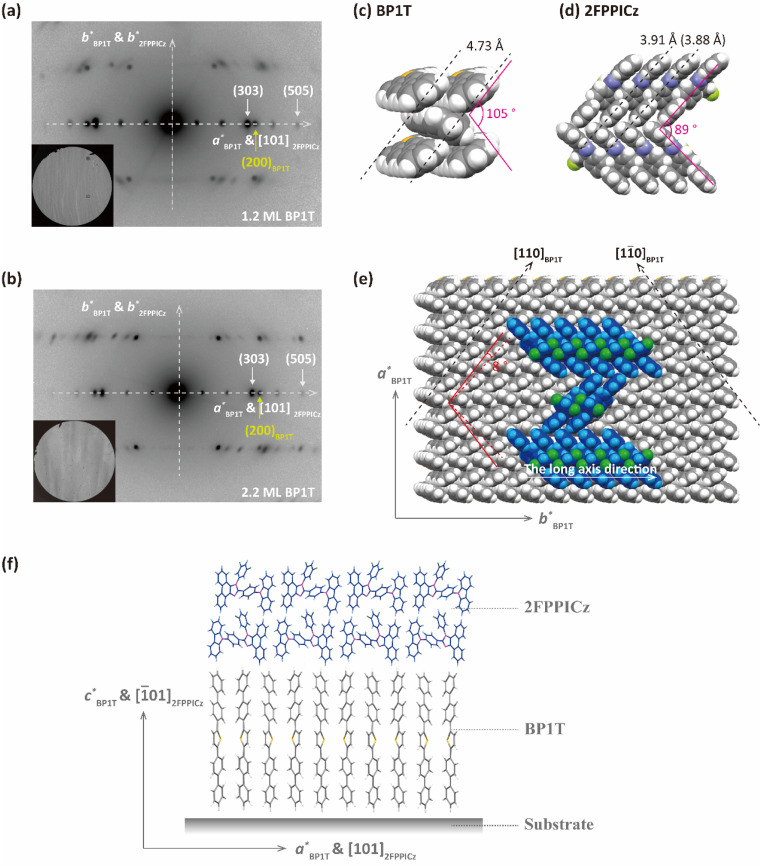
Epitaxial orientation of 2FPPICz on BP1T. (a and b) Selected area electron diffraction (SAED) patterns of 20 nm 2FPPICz thin film on 1.2-monolayer BP1T (a) and 2.2-monolayer BP1T (b), and the bottom left insets are the corresponding electron micrographs. (c and d) Crystal structures of BP1T and 2FPPICz molecules as viewed along the *c* axis and the [1̄01] projection, respectively. (e) The schematic diagram of the 2FPPICz epitaxial grown on BP1T. (f) Molecular packing model viewed from the direction parallel to the substrate.

The epitaxy relationship between 2FPPICz and the BP1T substrate was studied with transmission electron microscopy (TEM) and selected area electron diffraction (SAED) characterization. As exhibited in Fig. 3a and b, just one set of 2FPPICz in-plane orientation with the b* axis of 2FPPICz parallel to the b* axis of BP1T was revealed on both BP1T monolayer and double-layer thin film. The epitaxial relationship is as follows: (1̄01)_2FPPICz_//(001)_BP1T_, [101]_2FPPICz_//[100]_BP1T_, and [010]_2FPPICz_//[010]_BP1T_. Comparing the SAED pattern and the TEM morphology image, it can be obtained that the long axis of the 2FPPICz crystal corresponds to the b* axis of 2FPPICz and BP1T, and the short axis of the 2FPPICz crystal corresponds to the [101]_2FPPICz_ direction, that is, the a* axis direction of BP1T. The lattice mismatching between the (1̄01)_2FPPICz_ and (001)_BP1T_ planes was calculated and the interplanar spacing was presented in Table 1. On BP1T monolayer and double-layer thin film, the mismatching along a* of BP1T is 3.36% and 1.82%, respectively. It can be seen that the mismatching on the BP1T double-layer is smaller than that on the monolayer along the direction of a* axis of BP1T. This is the reason that the width of 2FPPICz crystals on the BP1T double-layer is greater than that on the monolayer as mentioned above. In other words, it is more conducive to the growth of large-size 2FPPICz crystalline thin film on the BP1T double-layer, which is beneficial to reduce film defects and improve the continuity of thin films.^21^

**Table 1:** The interplanar spacing of BP1T and 2FPPICz^a^

	BP1T (monolayer)	BP1T (double-layer)	2FPPICz (on BP1T monolayer)	2FPPICz (on BP1T double-layer)
d _(100)_ (Å)	7.50 ± 0.04	7.56 ± 0.04	—	—
d _(101)_ (Å)	—	—	12.92 ± 0.04	12.83 ± 0.04

a [Mismatching% = (|3d(101)_2FPPICz_ − 5d(100)_BP1T_|)/(5d(100)_BP1T_)].

Through the above measurement and analysis, the epitaxial mechanism of 2FPPICz on BP1T can be obtained. The protrudent hydrogen atoms of BP1T form the [110] and [11̄0] surface channels as the black dashed arrow displayed in Fig. 3e. The width of the channels is 4.73 Å as shown in Fig. 3c. From the reported single crystal data, the 2FPPICz molecules have two different conformations.^17^ And the distance between the parallel molecules of each conformation is different, which are 3.91 Å and 3.88 Å, respectively, as depicted in Fig. 3d. Both of them are smaller than the channel distance. As a result, each conformation of 2FPPICz molecules can be aligned into the channels. At the same time, due to the decisive effect of the lattice matching with the substrate on the growth process, the long axes of 2FPPICz molecules are finally arranged at an angle of 8° to the [110]_BP1T_ and [11̄0]_BP1T_ channels, respectively. The π–π stacking direction of 2FPPICz molecules corresponds to the long axis direction of the strip-like crystal. In order to more intuitively represent the orientation of molecules, we displayed the molecular packing model viewed from the direction parallel to the substrate as shown in Fig. 3f. BP1T molecules are arranged perpendicular to the substrate. The long axis of the 2FPPICz molecule is parallel to the substrate, which is the advantageous orientation for the light output of OLEDs.^22,23^

**Fig. 4:**
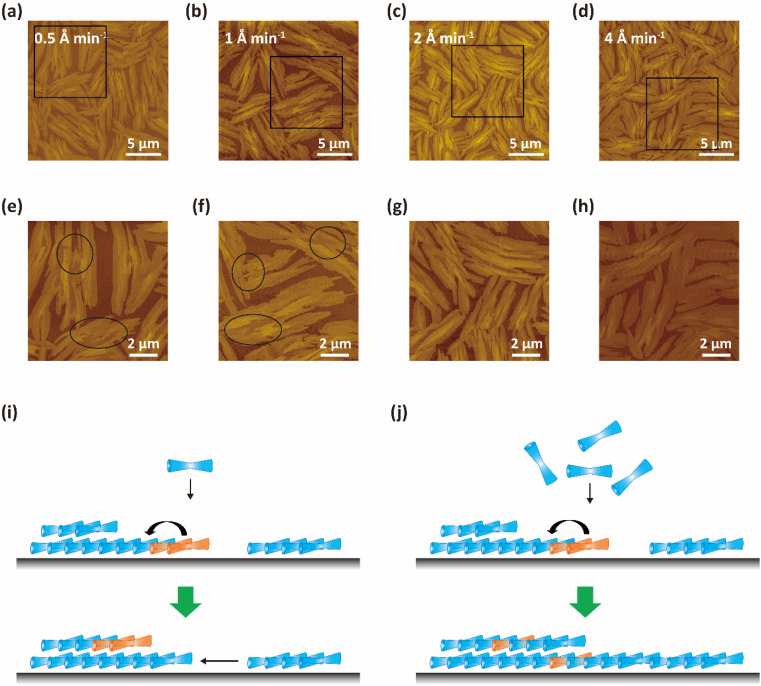
Morphology of 2FPPICz crystalline thin films with different growth rates on BP1T. (a–d) The AFM images of 2 nm thick 2FPPICz thin films on 2.2-monolayer BP1T at the growth rates of 0.5, 1, 2, and 4 Å min^−1^, respectively. (e–h) The corresponding zoomed-in images of the regions marked in (a–d). (i) Schematic diagram with the growth rate less than the transition rate. (j) Schematic diagram with the growth rate greater than the transition rate.

Based on the above results, it can be concluded that the formation of 2FPPICz thin films with wider crystal stripes on BP1T double-layer is more conducive due to a smaller lattice mismatch. Thus, we investigated the growth behavior of the 2FPPICz initial ultrathin films grown on BP1T double-layer for the purpose of obtaining high-quality crystalline films. The AFM images of 2 nm thick 2FPPICz thin film grown on 2.2-monolayer BP1T at different growth rates are shown in Fig. 4. When the growth rates were 0.5 Å min^−1^ (Fig. 4a) and 1 Å min^−1^ (Fig. 4b), the 2FPPICz crystals showed obvious liquid-like morphology and dewetting behavior as depicted in the black solid ellipses in Fig. 4e and f. It is thermodynamically metastable for monolayer film and will transform into multilayer films that are thermodynamically stable as displayed in Fig. S4† according to the measured film thickness. There is a greater velocity of transition than growth at this time. As displayed in Fig. 4i, when the growth rate is smaller than the transition rate, the molecules in the deposited monolayer film have enough time to evaporate and condense into the more stable double-layer nuclei before the subsequent molecules are deposited, or the double-layer nuclei will directly absorb molecules from monolayer films. So there are easily forming discontinuous regions between crystal domains. Therefore, it is hard to acquire large-size and continuous 2FPPICz crystalline thin films. When the growth rate was increased to 2 Å min^−1^, there was an apparent reduction in dewetting behavior as displayed in Fig. 4c. When the growth rate reached 4 Å min^−1^, the dewetting phenomenon almost disappeared as described in Fig. 4d. At this time, the rapid growth of the film makes it too late for the deposited molecules to diffuse, or there are the subsequent molecules quickly fill in after the diffusion of the deposited molecules as shown in Fig. 4j. Under this condition, it is practicable to prepare high-quality and continuous 2FPPICz crystalline thin films. Then, 2FPPICz thin films with different thicknesses were prepared at the growth rate of 4 Å min^−1^, and the transformation behavior of the films was observed before and after annealing at 102 °C for 2 hours by AFM, as shown in Fig. S5.† When the thickness of 2FPPICz thin film is 2.0 nm, it can be found by comparing the morphology before and after annealing that the dewetting behavior can be observed after annealing (Fig. S5a–c†). It shows that the initial ultrathin film is still in the thermodynamically metastable state at this time, and when sufficient activation energy is provided, the monolayer film will still transform into multilayer films. This phenomenon is similar to the previous reports and it can be summarized into three growth mechanisms, including direct absorption of molecules from monolayer films, migration of small molecular clusters, and quasi-Ostwald ripening.^24–26^ However, when the thickness was increased to 4.0 nm, the morphology of the 2FPPICz thin film did not change significantly after annealing (Fig. S5d and e†). Thus, the thin film is in a thermodynamic stable state at this moment. It further illustrates that the transformation of the monolayer film requires certain conditions and time. The actual growth process of the film is a continuous growth process, and what we observe is actually a kinetically controlled thin film growth process with a growth rate greater than the transition rate. Therefore, high-quality crystalline films can be obtained by controlling the kinetic factors of film growth.

**Fig. 5:**
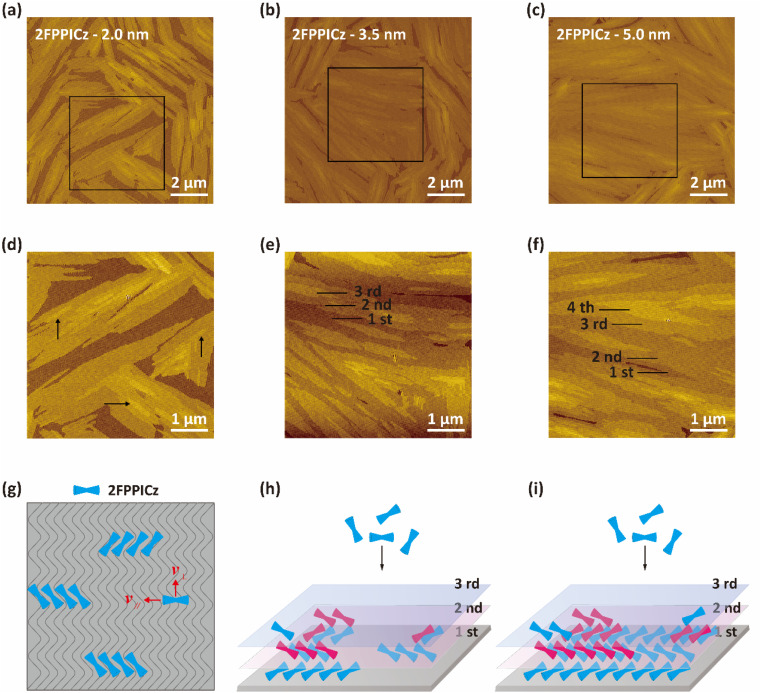
Morphologies of 2FPPICz with different thicknesses on BP1T. (a–c) AFM images of different thicknesses of 2.0 nm (a), 3.5 nm (b), and 5.0 nm (c) 2FPPICz grown on 2.2-monolayer BP1T. (d–f) The corresponding zoomed-in images of (a–c). (g–i) Schematic diagrams of the 2FPPICz thin film growth process.

The preparation and growth of high-quality continuous organic thin films with low defects, large-size and highly ordered domains are essential to create high-performance devices.^7,27,28^ More work needs to be carried out to understand the formation of the organic thin films, so as to further improve the device's performance. Thus, to study the growth process of the 2FPPICz crystalline thin films, we have grown different thicknesses films of 2FPPICz on 2.2-monolayer BP1T as shown in Fig. 5. At the early stage of the thin film growth, based on the sufficient activation energy provided by the substrate temperature, the 2FPPICz molecules can diffuse on the substrate and then start to align and grow until reaching a proper position where is the lowest potential energy position because of existing lattice matching relationship with BP1T.^29^ When a critical number of molecules are meeting, they deposit to form stable strip-like crystalline nuclei as the black arrows exhibited in Fig. 5d. With the thickness increasing, the new molecules still form new crystalline nuclei and incorporate into the current crystalline nuclei. When the thickness is increased to 3.5 nm, the first layer of 2FPPICz domains gradually meets and coalesces to almost completely covered the induction layer as shown in Fig. 5b. At the same time, the second and third islands continue to grow and expand as displayed in Fig. 5e. With the deposition of molecules, subsequent molecular layer islands also gradually grow and merge to form continuous films as exhibited in Fig. 5c. At this point, the second layer of the film has almost completed growth and the nuclei of the subsequent layer form and growth at the same time as exhibited in Fig. 5f. The growth process can be depicted by the process shown in Fig. 5g–i. Epitaxial molecular diffusion on surfaces containing ordered geometric channel structures is anisotropic, as shown in Fig. 5g, where the velocity in the vertical direction is much smaller than that in the parallel direction.^30,31^ This diffusion advantage can cause anisotropy in the nucleation and growth of epitaxial molecules, leading to the formation of oriented crystals. As the epitaxial molecules continue to be deposited, the monolayer crystal nuclei continue to grow, and the second and third layer crystal nuclei are simultaneously formed as shown in Fig. 5h. Despite the fact that multiple layers of islands grow simultaneously, the former thin film always completes the growth before the subsequent thin film as displayed in Fig. 5i, which provides favorable factors for the formation of multilayer continuous crystalline films. The preparation of low-defect and highly oriented light-emitting layers is the basis for subsequent functional layer growth and fabrication of high-performance C-OLED devices. Moreover, large-size continuous crystalline films with different thicknesses can be prepared as depicted in Fig. S6a–c† to meet different application requirements.

From previous work,^13^ three absorption peaks were observed in the ultraviolet-visible (UV-vis) absorption spectrum of the 2FPPICz crystalline film at 335 nm, 350 nm, and 368 nm, respectively (Fig. S7a,† black line). The absorption peaks are due to the transition from S_0_ to different S_1_ states within the 2FPPICz molecules. Upon excitation wavelength with 340 nm, the 2FPPICz crystalline film exhibited a maximum emission peak at 405 nm with two shoulder peaks(Fig. S7a,† red line). To examine the relationship between the lifetime and the three fluorescence emission peaks, we tested the time-resolved photoluminescence (Fig. S7b†) and transient decay curves (Fig. S7c†) of 40 nm-thick 2FPPICz crystalline thin film. The time-resolved photoluminescence results indicate that the fluorescence spectrum of the 2FPPICz crystalline film shifts towards the red end of the spectrum as the gate time is increased from 3 ns to 9 ns. This finding suggests that the surface states of the 2FPPICz crystalline film have various energy levels. Furthermore, the transient decay curves of the 2FPPICz crystalline film demonstrate that different lifetimes at different emission wavelengths (*E*_m_ = 385 nm − 405 nm − 440 nm, *τ* = 0.84 ns − 1.02 ns − 1.94 ns), which is consistent with the time-resolved photoluminescence results. All the fluorescence lifetime decay curves can be well fitted by bi-exponential decay (Table S1†).

## Conclusions

In a word, 2FPPICz crystalline thin film grown on BP1T layer is the key layer and host matrix in C-OLEDs. Thus, further understanding the 2FPPICz crystalline thin film growth on BP1T induction layer is critical for further development of the C-OLEDs. In this paper, we have investigated the morphology of 2FPPICz crystals on different thicknesses of BP1T thin films. 2FPPICz crystals can epitaxial growth to form strip-like crystals on both BP1T monolayer and double-layer. But on BP1T double-layer, 2FPPICz crystals have a smaller root-mean square roughness and a larger width than those on BP1T monolayer. This is due to the smaller lattice mismatch on the BP1T double-layer obtained in the SAED measurement and analysis. And these experiments led to the epitaxial model of 2FPPICz on BP1T substrate, that is, the molecular long axis of 2FPPICz is parallel to the substrate, too. In addition, the study of film growth kinetics found that the initial 2FPPICz ultrathin films are in a thermodynamically metastable state. When the growth rate is lower than the transition rate, the initial ultrathin film will exhibit a dewetting behavior, which is not conducive to form continuous thin films. As a result, high-quality continuous crystalline thin films can only be prepared when the growth rate is greater than the transition rate. Under this condition, 2FPPICz multilayer films grow simultaneously, but the former layer of thin film always preferentially completes its growth. Based on that, different thickness large-size continuous crystalline thin films can be prepared according to application requirements. These studies on 2FPPICz crystalline thin film will provide guidelines in optimizing WEG process for creating high-performance C-OLEDs.

## Experimental section

### Materials

BP1T and 2FPPICz were synthesized using the previously reported method.^32,33^ Both of them were purified twice by thermal gradient vacuum sublimation before usage.

### Fabrication and characterization of films

Before usage, the Si/SiO_2_ substrate needs to be cleaned. The absorbent cotton was moistened with acetone to wipe the Si/SiO_2_ substrate first. Then acetone, ethanol, and deionized water were used to rinse the substrate in sequence. Following three rounds of rinsing, the substrate was desiccated with high-purity nitrogen. After that, the Si/SiO_2_ substrate was fixed with clamps and was put into the vacuum chamber with pressure below 10^−4^ Pa. At the substrate temperature of 102 °C, the BP1T thin films were deposited on the Si/SiO_2_ substrate at the evaporation rate of about 2–3 Å min^−1^ and 2FPPICz thin film was deposited on the BP1T thin films at the evaporation rate of about 0.5–4 Å min^−1^. The evaporation rate and the thickness could be monitored with a quartz-crystal microbalance. The morphologies of the thin films were observed using an atomic force microscope (AFM SPI 3800/SPA, 300 HV, Seiko Instruments Inc., Japan) with tapping mode. A thin-film diffractometer (Bruker D8 Discover) with Cu Kα radiation (*λ* = 1.54056 Å) was used to collect the out-of-plane X-ray diffraction (XRD) data in the locked coupled mode. And a Rigaku SmartLab X-ray diffraction instrument was used to acquire the in-plane XRD patterns (*λ* = 1.54056 Å). Time-resolved photoluminescence spectra and transient decay curves were obtained by Edinburgh Instrument FLS980 spectrometer.

### Fabrication and characterization of TEM samples

First of all, using an ETD-800C type small thermal evaporation coating instrument (Vision Precision Instruments Inc., China), a carbon film was vacuum deposited on the surface of the thin films deposited on the Si/SiO_2_ substrate as the supporting layer to prevent the thin films from shattering during subsequent operations. After that, taking advantage of the difference in hydrophobicity and expansion coefficient between the sample and the silica, the carbon film-fixed sample was separated from the Si/SiO_2_ surface and floated on the surface of the 10% HF solution. In the end, the thin film with carbon coating was captured with a 400 mesh copper grid and then placed on a filter paper to absorb water and dried. The selected area electron diffraction (SAED) patterns were acquired with a transmission electron microscope (JEOL JEM-1011) at 100 kV, and the corresponding electron micrographs were obtained under bright field conditions.

## Author contributions

F. Z. and D. H. Y. initiated and designed the research. D. L. carried out the growth and characterization of crystalline thin films. All authors discussed the results, analyzed the data and prepared the manuscript. F. Z., D. H. Y. supervised the project.

## Conflicts of interest

There are no conflicts to declare.

## Supplementary Material

RA-013-D3RA03095D-s001
