# Supplementary material for: Crystalline organic thin films for crystalline OLEDs (II): weak epitaxy growth of phenanthroimidazole derivatives
Source: RSC Adv. 2023 May 23;13(23):15586–93. doi: 10.1039/d3ra03095d (PMC10203860; doi:10.1039/d3ra03095d)
Supplement: RA-013-D3RA03095D-s001 [file RA-013-D3RA03095D-s001.pdf]

Electronic Supplementary Material (ESI) for RSC Advances.
This journal is © The Royal Society of Chemistry 2023

Supplementary Information

Crystalline organic thin films for crystalline OLEDs (II): weak epitaxy growth of phenanthroimidazole derivatives

Dan Liu[1,2], Feng Zhu[1,2*], Donghang Yan[1,2]

[1]State Key Laboratory of Polymer Physics and Chemistry, Changchun Institute of Applied Chemistry, Chinese Academy of Sciences, Changchun 130022, China.

[2]School of Applied Chemistry and Engineering, University of Science and Technology of China, Hefei 230026, China.

*Corresponding author. Email: zhufeng@ciac.ac.cn

Contents:

1. **Morphologies of BP1T films grown at different substrate temperatures**
2. **Morphologies of different thicknesses BP1T**
3. **Width of 2FPPICz strip-like crystal on BP1T**
4. **Height of 2FPPICz crystals on double-layer BP1T**
5. **Morphologies of different thicknesses 2FPPICz before and after annealing**
6. **Morphologies of different thicknesses 2FPPICz on BP1T**
7. **Optical properties of 2FPPICz crystalline film**

1. Morphologies of BP1T films grown at different substrate temperatures

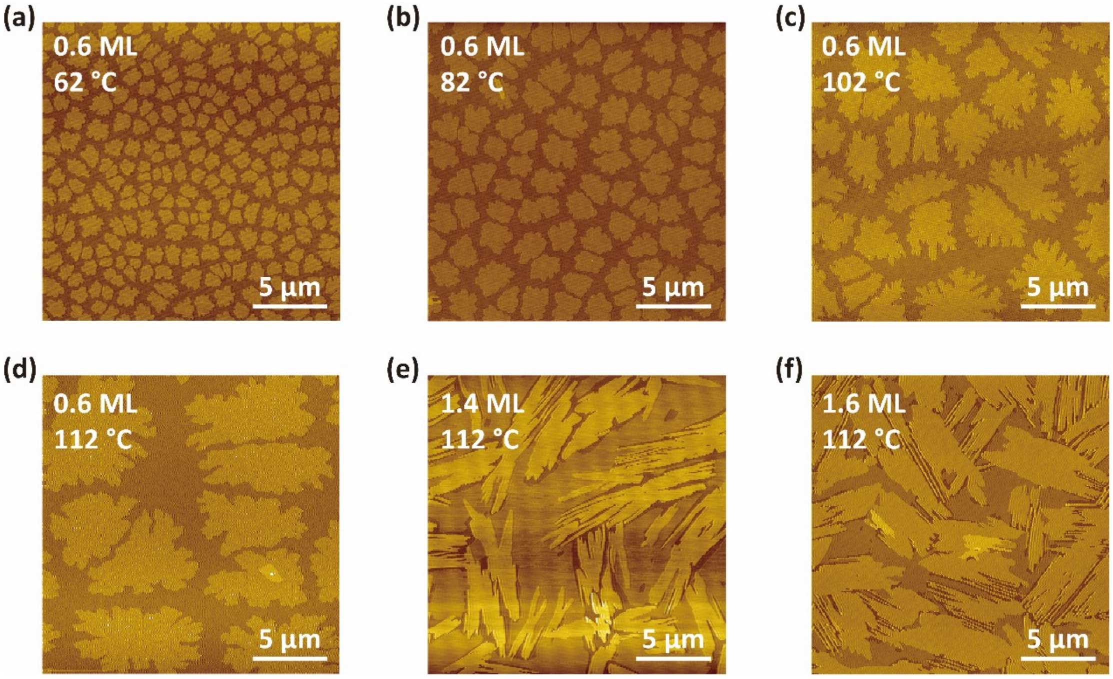

Fig. S1 AFM images of BP1T films grown at different substrate temperatures. (a-d) 0.6 ML BP1T grown at 62 °C (a), 82 °C (b), 102 °C (c), and 112 °C (d). (e-f) 1.4 ML (e) and 1.6 ML (f) BP1T grown at 112 °C.

2. Morphologies of different thicknesses BP1T

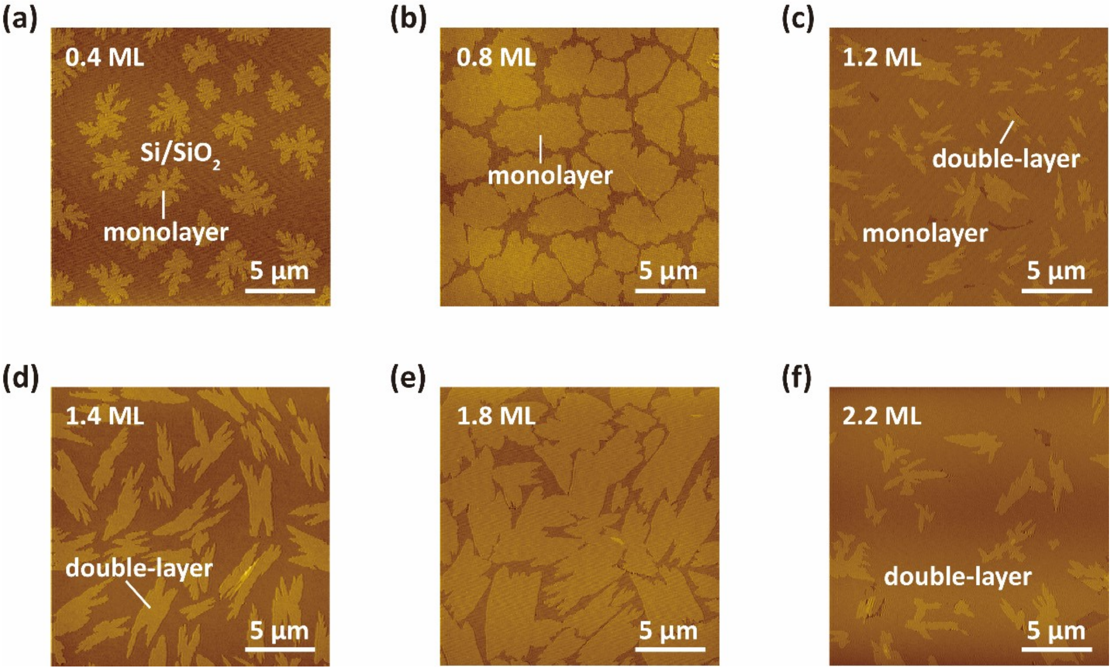

Fig. S2 AFM images of 0.4 (a), 0.8 (b), 1.2 (c), 1.4 (d), 1.8 (e), and 2.2 (f) monolayer BP1T films at the substrate temperature of 102 °C.

3. Width of 2FPPICz strip-like crystal on BP1T

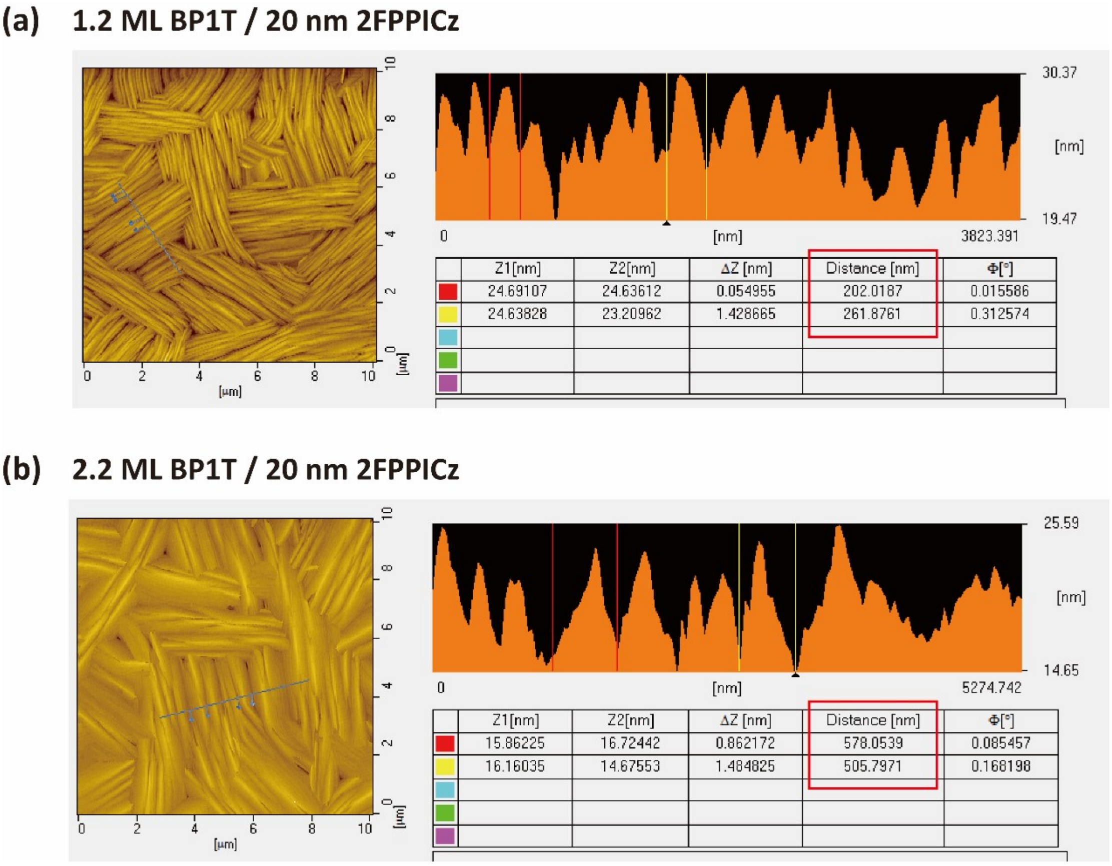

Fig. S3 (a-b) Width of 2FPPICz strip-like crystals grown on 1.2 (a) and 2.2 (b) monolayer BP1T.

4. Height of 2FPPICz crystals on double-layer BP1T

(a)

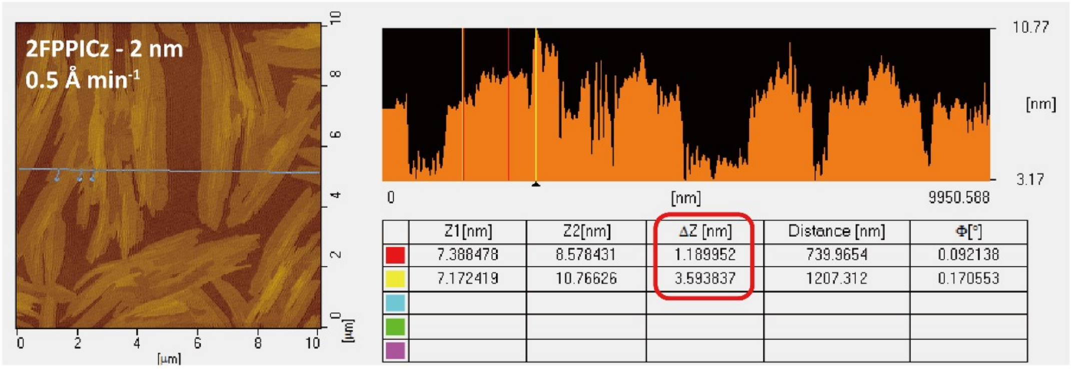

Fig. S4 Height map of 2-nm thick 2FPPICz thin film deposited on 2.2-monolayer BP1T.

5. Morphologies of different thicknesses 2FPPICz before and after annealing

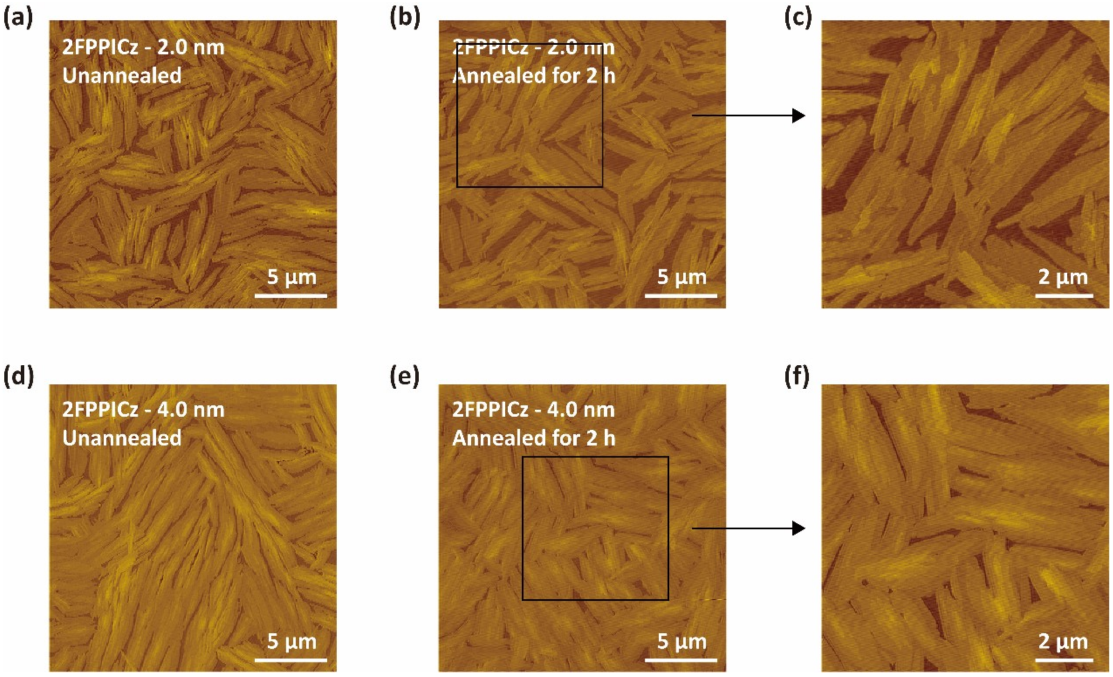

Fig. S5 (a and b) AFM images of unannealing (a) and annealing for 2 hours (b) 2.0-nm thick 2FPPICz thin film. (d and e) AFM images of unannealing (d) and annealing for 2 hours (e) 4.0-nm thick 2FPPICz thin film. (c and f) The corresponding zoom images of (b) and (e).

6. Morphologies of different thicknesses 2FPPICz on BP1T

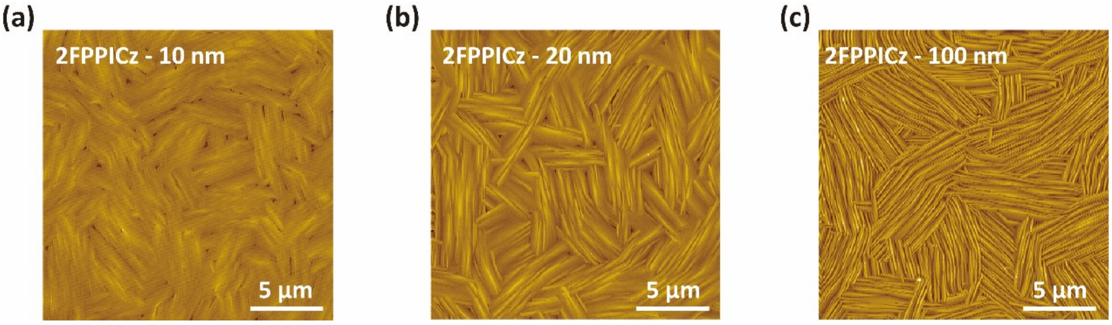

Fig. S6 Morphologies of 2FPPICz with different thicknesses on BP1T. The AFM images of different thicknesses of 10 nm (a), 20 nm (b), and 30 nm (c).

7. Optical properties of 2FPPICz crystalline film

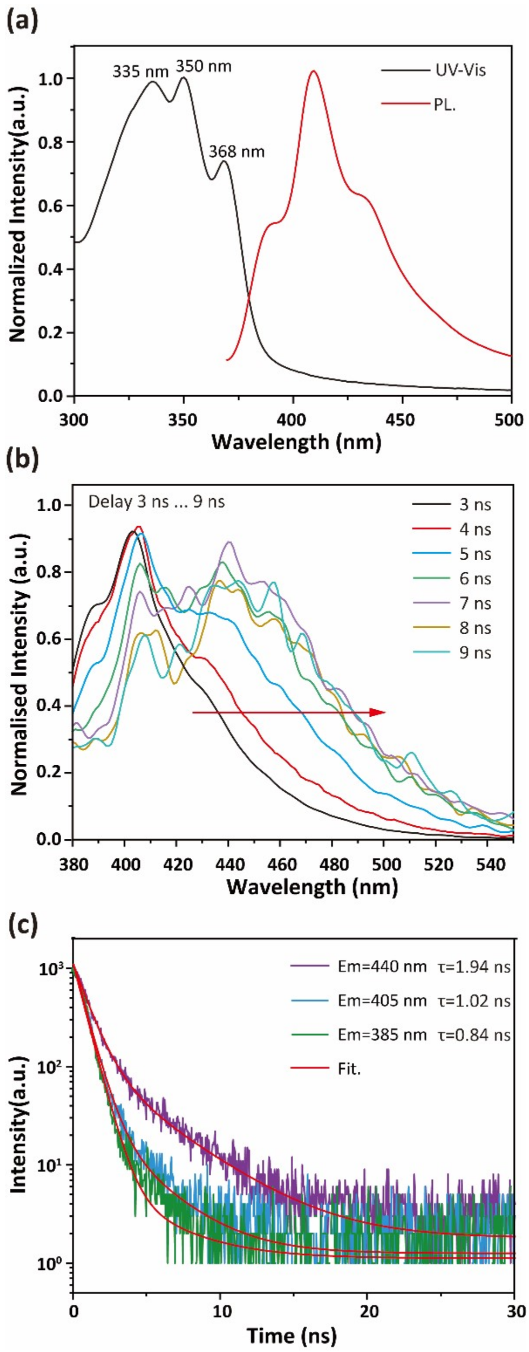

Fig. S7 Optical properties of 2FPPICz crystalline film. (a) Normalized ultraviolet-visible (UV-vis) absorption and photoluminescence (PL) spectrum of 2FPPICz crystalline thin film from [J. Mater. Chem. C, 2021, 9, 2236-2242]. (b) Time-resolved photoluminescence spectra of 40 nm-thick 2FPPICz crystalline thin film. (c) Transient decay curves of 40 nm-thick 2FPPICz crystalline thin film.

Table S1 Fluorescence lifetimes (τ) of 2FPPICz crystalline film depend on wavelength.

Em (nm)	A_1	τ_1 (ns)	A_2	τ_2 (ns)
385	1148.42	0.74	8.7	3.56
405	1032.68	0.75	36.49	2.98
440	1025.86	0.97	122.11	3.94
